# The Role of Susceptibility in the Association Between Exposures and Occupational Contact Dermatitis: A Scoping Review

**DOI:** 10.1111/cod.70030

**Published:** 2025-09-12

**Authors:** Renate Juścikowski, Pieter Coenen, Sanja Kezic, Damien M. McElvenny, Faridi S. Jamaludin, Henk F. van der Molen, Jan L. Hoving

**Affiliations:** ^1^ Amsterdam UMC, University of Amsterdam Public and Occupational Health Amsterdam the Netherlands; ^2^ Amsterdam Public Health Clinical Occupational and Medicine and Occupational Disease Amsterdam the Netherlands; ^3^ Amsterdam UMC, Vrije Universiteit Amsterdam Public and Occupational Health Amsterdam the Netherlands; ^4^ Amsterdam Public Health Societal Participation and Health Amsterdam the Netherlands; ^5^ Institute of Occupational Medicine Edinburgh UK; ^6^ Centre for Occupational and Environmental Health, University of Manchester Manchester UK; ^7^ Medical Library Academic Medical Center (AMC) Amsterdam UMC Location University of Amsterdam Amsterdam the Netherlands

**Keywords:** Eczema, Exposure assessment, Hazardous substances, Individual susceptibility factors, Irritant and allergic CD, Occupational disease, Risk assessment, Scoping review

## Abstract

The objective of this review is to identify individual susceptibility factors and determine their role in the association between work‐related exposures and contact dermatitis (CD). A scoping review was conducted using Medline, Embase, and CINAHL. Cohort and case‐control studies were included for all types of CD, and cross‐sectional studies for allergic contact dermatitis (ACD). In the absence of meta‐analysis, we drew qualitative inferences summarising the findings. Twenty‐one studies, primarily cross‐sectional (*n* = 18), investigated how 44 individual susceptibility factors influenced associations between 53 work‐related exposures (from six exposure categories) and CD. These factors were grouped into five categories: demographic, socioeconomic, host‐related intrinsic factors, lifestyle, and exposures outside work. The factors variously mitigated, amplified, or had no effect on the exposure‐CD association. The role of individual susceptibility factors in work‐related exposure‐CD associations remains underexplored and inconclusive. Determining their individual contributions is challenging, as studies often adjust for multiple factors, with inconsistent influence on the association. Age, sex, atopic history, hand eczema, smoking, and ethnicity may influence CD risk and should be considered in studies examining work‐related exposure‐CD associations. Further research is needed to clarify the role of individual susceptibility factors and guide effective prevention strategies for occupational CD.

## Graphical abstract

- Substantial evidence links work‐related exposures to occupational contact dermatitis, but worker responses vary.
- A total of 44 individual susceptibility factors and 53 work‐related exposures were identified.
- The impact of non‐work‐related susceptibility factors remains unclear.
- Factors such as age, sex, atopic dermatitis, hand eczema, smoking, and ethnicity should be considered.

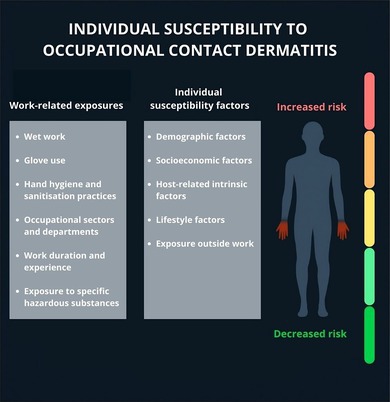

## 1 Introduction

Contact dermatitis (CD) is an inflammatory skin condition that results from direct skin contact with skin‐irritating factors or allergens [1]. The primary types of CD include irritant contact dermatitis (ICD) and allergic contact dermatitis (ACD) [2]. ICD is caused by direct damage to the skin barrier after exposure to physical and/or chemical agents, resulting in inflammation and activation of the innate immune system. ACD, on the other hand, is associated with type IV delayed‐type hypersensitivity, involving both innate and acquired immunity [2]. Common skin irritants include water (wet work), detergents, degreasing agents, and solvents, while key allergens include metals (e.g., nickel, cobalt, chromium), isocyanates, preservatives such as isothiazolinones, rubber, and epoxy resins [3, 4, 5]. When exposure to such irritants or allergens occurs in the workplace, resulting cases of ICD and/or ACD can be classified as occupational CD [6].

Occupational CD is the most prevalent occupational skin disease, accounting for 95% of all reported cases [3]. ICD constitutes approximately 80% of CD cases, while ACD accounts for the remaining 20%, although this varies by occupation [7]. Occupational CD places a significant burden on affected individuals and society, with annual healthcare and productivity costs exceeding $1 billion in the United States alone [2, 8]. Epidemiological studies estimate an annual incidence of 5–19 registered cases of occupational CD per 10,000 full‐time workers, with a 1‐year prevalence of around 10% and a lifetime prevalence of approximately 20% [7, 8, 9]. However, the true prevalence is likely much higher due to underreporting of mild cases, potentially by a factor of 20–50 [7]. Certain subgroups, particularly those in high‐risk occupations such as healthcare, hairdressing, and construction, are at increased risk of CD due to prolonged or intense exposure to irritants and allergens [4, 6, 10, 11].

Although work‐related exposures form a significant risk factor, individual susceptibility—defined in this review as the combination of non‐work‐related factors such as demographic, socioeconomic, personal and family history of atopic skin diseases, and environmental factors such as lifestyle choices and non‐occupational co‐exposures—has the potential to play a crucial role in the association between work‐related exposures and CD [12, 13]. Various modifiable and non‐modifiable susceptibility factors may explain why some individuals develop CD under similar exposures while others do not. Non‐modifiable factors include inherited traits, such as variations in genes that influence the skin barrier function and immune response, sex, ethnic background, and pre‐existing health conditions, with atopic dermatitis (AD) being the most significant, while modifiable factors involve lifestyle and environmental factors such as smoking, alcohol consumption, diet, physical activity, and co‐exposures at home [11, 12, 14]. Such factors have the potential to individually or collectively mediate, moderate, and/or confound the association between work‐related irritant/allergen exposures and CD [11, 12, 14, 15, 16]. Due to the different pathophysiology of ACD and ICD, non‐work‐related susceptibility factors might differ between the two types of CD [6]. However, there is some degree of overlap, particularly in factors related to innate immunity and skin barrier function.

Individual susceptibility has gained attention in recent decades, but it is often used interchangeably with terms like vulnerability or predisposing factors, leading to conceptual inconsistencies [6, 17]. Although some research has explored how non‐work‐related factors affect occupational CD risk, their influence on the association between work‐related exposures and CD remains poorly understood. To date, no reviews have specifically addressed the role of individual susceptibility in this association, indicating a significant gap in the literature. Given the definitional ambiguity and heterogeneity across studies, a systematic review is currently unfeasible. A scoping review, however, could offer a structured preliminary evaluation, enhancing understanding of individual susceptibility in occupational CD.

The objectives of this review were therefore to identify individual susceptibility factors and determine their role in the association between work‐related exposures and CD.

## 2 Methods

The protocol of this scoping review was published in the Open Science Framework (OSF), on August 1, 2024 [18]. Deviations from the protocol included expanding the study design from cross‐sectional studies on ACD to also include studies where the type of CD was undefined, but where both irritant and allergen exposures were present—indicating potential ICD and ACD. In addition to non‐work‐related susceptibility factors, we also reported work‐related factors, as studies often adjust for a combination of these two groups of factors. Reporting followed the Preferred Reporting Items for Systematic Reviews and Meta‐Analyses extension for Scoping Reviews (PRISMA‐ScR) [19].

### 2.1 Search Method

Conducted by a medical information specialist (FSJ) on February 20, 2024, in MEDLINE (Ovid), Embase (Ovid), CINAHL (Ebscohost), the literature search included both standardised keywords (e.g., MeSH terms) and free text terms such as “contact dermatitis/eczema,” “allergic/irritant dermatitis,” “occupational/work,” “occupational exposure,” “risk factors/assessment,” and “causality,” as outlined in Supporting Information S1. Retrieved citations were imported into Rayyan for screening, and duplicates were removed using automated and manual checks [20]. Reference lists of included papers were reviewed to identify additional potentially relevant publications.

### 2.2 Study Eligibility

Articles were included if they met the predefined PECO criteria and the following additional eligibility criteria: (1) samples included workers, apprentices, industries, or the general population with known work‐related exposure to irritants/allergens; (2) diagnosis of (contact) dermatitis (ICD and/or ACD) was performed clinically or via self‐reports; (3) full‐text articles reported quantified effect estimates (e.g., prevalence ratios (PR), odds ratios (OR), or hazard ratios (HR)) for non‐work‐related susceptibility factors that may mediate, moderate, or confound the association between a work‐related exposure and CD. This includes both unadjusted and adjusted estimates that account for these factors in the association between work‐related irritant or allergen exposures and CD; (4) retrospective or prospective cohort, case‐cohort, case‐control, case‐referent, or cross‐sectional studies (for ACD outcomes only, as risk factors are often investigated using registry data or patch tests); (5) publication in a peer‐reviewed journal; (6) the article was written in English or Dutch. See Supporting Information S1 for detailed selection criteria.

In the first round, one investigator (RJ) excluded clearly irrelevant articles, such as those with an incorrect population, outcome, or study design [21]. In the second round, titles and abstracts were screened independently by pairs of two investigators (RJ, PC, SK, DMM, HFM, JLH) using Rayyan [20]. Final eligibility was assessed by screening the full‐text articles in pairs using a similar process. In the absence of consensus, a third investigator was consulted.

### 2.3 Data Extraction and Analyses

Relevant study data were extracted by one investigator (RJ) and verified by a second independent investigator (PC, SK, DMM, HFM, JLH) using a predefined and piloted Excel spreadsheet. The following data were extracted: study characteristics (e.g., first author, study design, duration/year); study population (e.g., occupation, sample size, sex distribution); exposure assessment (e.g., exposure type, reported exposure, assessment tool); outcome assessment (e.g., reported outcome, type of assessment, assessment tool); the types and characteristics of the reported individual susceptibility factors, and both the unadjusted and adjusted (using individual susceptibility factors) effect estimates for the association between a work‐related exposure and CD. For consistency, “hand eczema” was labelled as “hand dermatitis” in the tables, and “(contact) dermatitis” was labelled as CD. In addition, the type of CD (irritant or allergic) was recorded in the tables if explicitly reported or determined based on the study's exposure and outcome assessment method. Cross‐sectional studies reporting only contact or hand dermatitis with both irritant and allergen exposures were categorised as mixed (indicating both ICD and ACD). In such cases, the assumed predominant type of CD was specified in the tables. The association for each exposure was reported with its 95% confidence interval, allowing comparison of the changes in magnitude and direction of effect estimates with and without adjustment for susceptibility factors.

Although we did not consider statistical pooling feasible due to substantial variability in exposures, outcomes, and factors, forest plots were generated for three major exposure categories: wet work, glove use, and hand hygiene and sanitisation practices. This allowed a visual assessment of the changes that occurred in the exposure–outcome association as a result of adjusting for non‐work‐related susceptibility factors and work‐related factors. We also calculated and tabulated the percentage change between unadjusted and adjusted associations within each study.

## 3 Results

### 3.1 Study Selection

**FIGURE 1:**
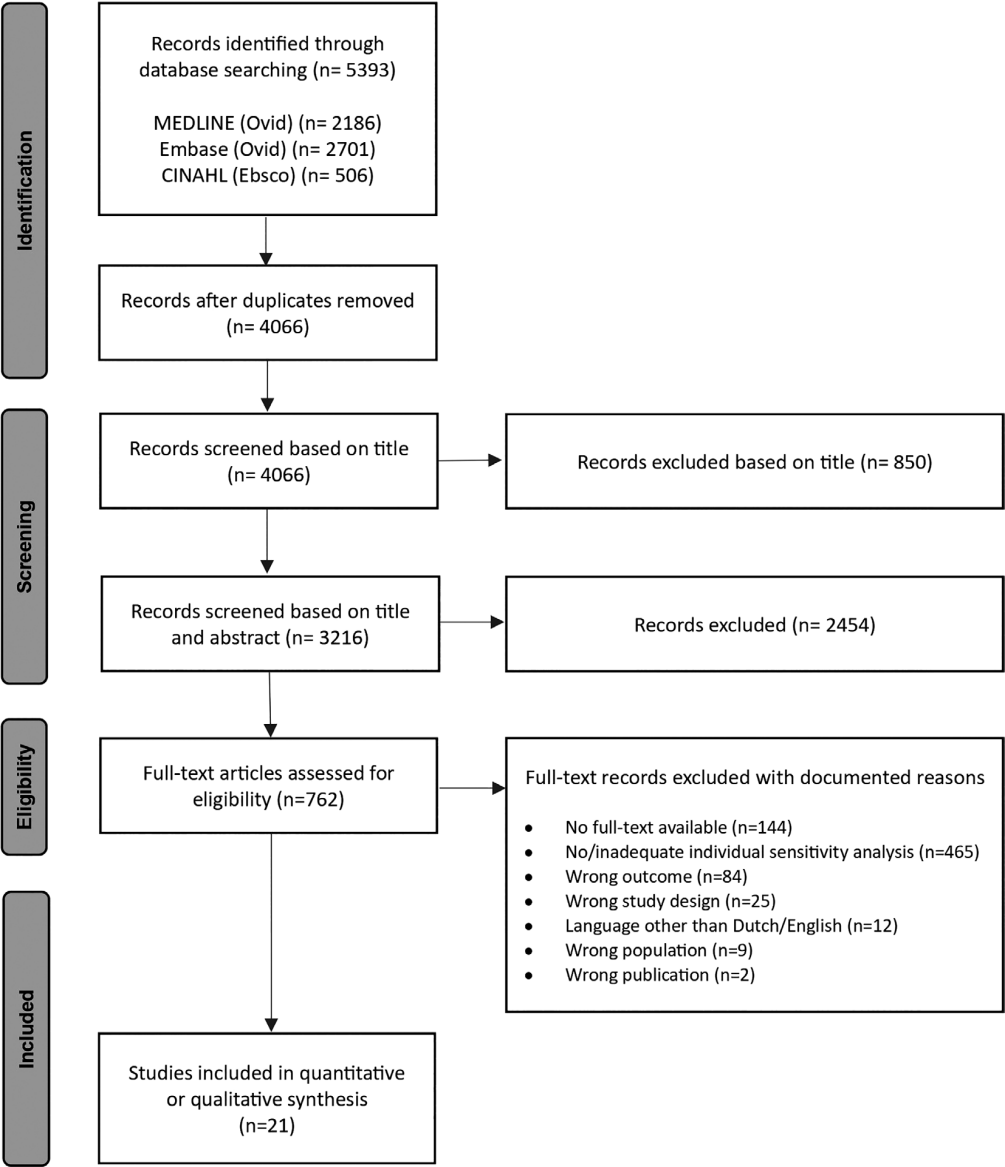
PRISMA flow diagram illustrating the search and selection process.

Following the removal of duplicates, a total of 4066 articles remained eligible for screening. Of these, 850 were excluded based on their title by one author (RJ), and 2454 were excluded after title and abstract screening, leaving 762 studies eligible for full‐text assessment. From this set, 741 studies were excluded, primarily due to the absence or inadequate analysis of susceptibility factors. Ultimately, 21 studies were included for data extraction and analysis. The full study selection process is illustrated in Figure 1.

### 3.2 Study Characteristics

**FIGURE 2:**
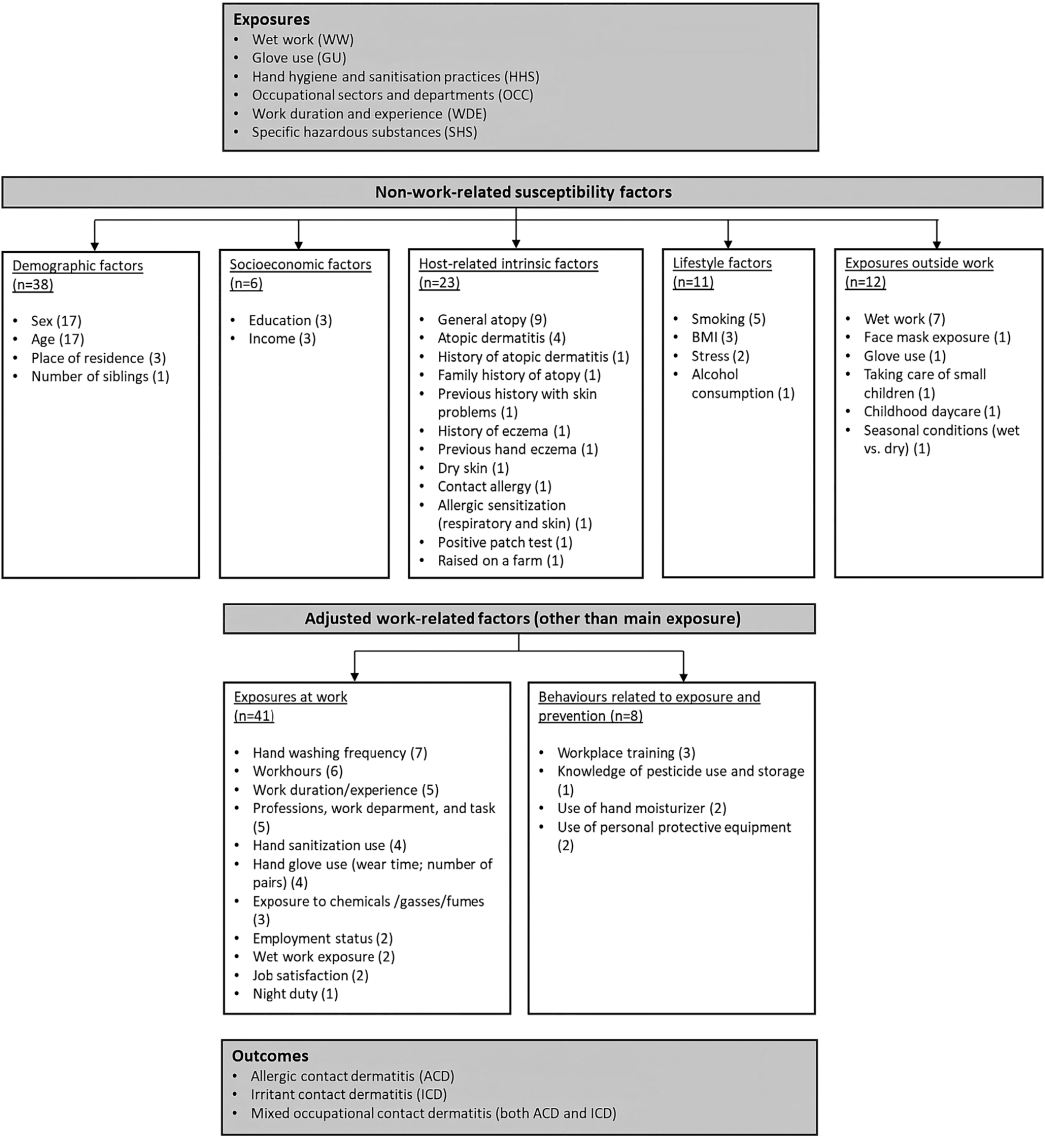
Overview of the identified exposures, outcomes, individual susceptibility factors, and adjusted work‐related factors, with the number of studies reporting each factor indicated in brackets.

The majority of included studies were conducted in Asian countries (*n* = 10) [22, 23, 24, 25, 26, 27, 28, 29, 30, 31], followed by European countries (*n* = 7) [32, 33, 34, 35] and African countries (*n* = 4) [36, 37, 38, 39, 40, 41, 42]. Most studies employed a cross‐sectional design (*n* = 18) [22, 23, 24, 25, 26, 27, 28, 29, 30, 31, 32, 33, 34, 35, 36, 37, 38, 40], while two studies used a case‐control design [41, 42] and one used a retrospective cohort design [39]. Of the 17 studies that examined occupations in specific sectors, the majority focused on the healthcare sector (*n* = 10) [22, 25, 26, 27, 28, 31, 33, 37, 38, 39], followed by agriculture (*n* = 2) [23, 34], and one study each on cleaning [35], automotive [32], hairdressing [24], construction [42], and furniture manufacturing [30]. Most studies reported either (hand) dermatitis or eczema (*n* = 15) [23, 24, 25, 26, 27, 28, 29, 31, 36, 37, 38, 40] or CD (*n* = 6) [22, 30, 32, 33, 34, 35], with mixed outcomes of ICD and ACD in 18 studies [22, 23, 24, 25, 26, 27, 28, 29, 30, 31, 32, 33, 34, 35, 36, 37, 38, 40]. One study specifically focused on ACD, identifying epoxy resins as a relevant contact allergen, with the hand being the primary site of exposure and manifestation [42]. An overview of the study characteristics is presented in Table 1. A total of 53 work‐related exposures contributing to CD were reported across diverse occupational settings, activities, and practices, including variations of (1) wet work exposure, (2) glove use, (3) hand hygiene and sanitisation practices, (4) occupational sectors and departments, (5) work duration and experience, and (6) exposure to specific hazardous substances. Figure 2 outlines the exposures, non‐work‐related factors, adjusted work‐related factors, and outcomes.

**TABLE 1:** Summary of the study characteristics of the included studies.

References	Study design	Occupation of exposed and controls		Sample size		Outcome	Outcome assessment	IS factors (*n*)
		Occupation exposed (median age in years (SD); % female)	Occupation controls (median age in years (SD); % female)	Cases (*n*)	Controls (*n*)			
Alluhayyan et al. [22] Saudi‐Arabia	Cross‐sectional	Healthcare workers (34.0 years (±9.0); 66.7%)	NA	408	NA	CD, classified as mixed^a^	NOSQ‐2002 questionnaire (self‐reported).	2
Ahn et al. [23] Vietnam	Cross‐sectional	Farmers, household family members in Huong Liet and Long Bien commune (47.9–44.7 years (NR); 75.0%–86.0%)	NA	592	NA	Dermatitis, classified as mixed (primarily ICD)^a^	NOSQ‐2002‐based questionnaire (interview) and dermatological examination.	7
Attwa et al. [32] Egypt	Cross‐sectional	Car repair workers: organic solvents, oils, rubber, nickel, metalworking fluids (33.7 years (±11.2); 0%)	Booksellers (32.8 years (±6.7); 0%)	87	76	CD, classified as mixed (40.0% ACD, 60.0% ICD)^a^	NOSQ‐2002‐based questionnaire (interview) followed by dermatological examination and patch test.	3
Brands et al. [36] The Netherlands	Cross‐sectional	General population (≥ 18 years) (55.8 years (±12.2); 60.3%)	NA	57 046	NA	HD, classified as mixed (primarily ICD)^a^	Questionnaire based on the NOSQ‐2002 (self‐reported).	5–9
Hajaghazadeh et al. [24] Iran	Cross‐sectional	Hairdressers (33.2 years (±7.2); 100.0%)	NA	385	NA	HD, classified as mixed (mainly ICD)^a^	NOSQ‐2002‐based questionnaire and photographic validated guide (interview).	2
Hamnerius et al. [38] Sweden	Cross‐sectional	Healthcare workers (46.0 years (NR); 83.0%)	NA	5094	NA	HD, classified as mixed (primarily ICD)^a^	An internet‐based questionnaire (Sunset Survey) (self‐reported).	5
Hamnerius et al. [37] Sweden	Cross‐sectional	Hospital employees (45.9 years (±12.0); 87.0%)	NA	9051	NA	HD, classified as mixed (primarily ICD)^a^	Electronic questionnaire (Relationwise Survey Solution) (self‐reported).	2–7
Huang et al. [25] China	Cross‐sectional	Healthcare workers (32.5 years (±7.9); 84.5%)	NA	521	NA	HD, classified as mixed (primarily ICD)^a^	NOSQ‐2002 questionnaire (self‐reported).	7
Lee et al. [26] Korea	Cross‐sectional	Hospital nursing staff (30.8 years (NR); 97.1%)	NA	525	NA	HD, classified as mixed (primarily ICD)^a^	An unidentified questionnaire (self‐reported) followed by a patch test.	8
Lund et al. [41] Denmark	Case‐referent	Various patients with HD (38.7 years (NR); 63.7%)	Various patients with facial eczema (40.7 years (NR); 81.0%)	11 706	5499	HD (primarily ICD)	Dermatological examination recorded in national database of contact allergy.	4–9
Meding et al. [39] Sweden	Retrospective Cohort	Dental technicians (46.0 years (NR); 55.8%)	Various population controls (45 years (NR); 56.9%)	1210	1316	HD	An unidentified questionnaire (self‐reported).	1
Mekonnen et al. [33] Ethiopia	Cross‐sectional	Healthcare workers (22.6 years (±6.3); 47.6%)	NA	422	NA	CD, classified as mixed (primarily ICD)^a^	NOSQ‐2002 questionnaire (interview)	11
Minamoto et al. [27] Japan	Cross‐sectional	Dentists, hygienists, technicians, assistants, receptionists (37.3 years (12.4); 80.3%)	NA	528	NA	HD, classified as mixed (primarily ICD)^a^	A NOSQ‐2002‐based questionnaire (self‐reported) followed by a patch test.	10
Rönmark et al. [40] Sweden	Cross‐sectional	General adult population (ages 16–75) (50.4 years (±15.4); 54.0%)	NA	1172	NA	Dermatitis, classified as mixed^a^	An unidentified questionnaire (self‐reported).	8
Smith et al. [28] Japan	Cross‐sectional	Nurses (various departments) (27.4–34.9 years (±5–5 – 10.2); 100.0%)	NA	350	NA	HD, classified as mixed (primarily ICD)^a^	An unidentified questionnaire (self‐reported).	7
Spee et al. [42] Germany	Case control	Construction workers with epoxy allergy (41.5 years (NR); NR)	Construction workers without epoxy allergy (41.0 years (NR); NR)	179	151	ACD	A diagnostic patch test and questionnaire based on Smit et al. and Jungbauer et al.	4
Tamene et al., 2021 [34] Ethiopia	Cross‐sectional	Farm labourers (28.8 years (±5.1); 0%)	NA	578	NA	CD, classified as mixed (primarily ACD)^a^	NOSQ‐2002 questionnaire (interview)	7
Techasatian et al. [29] Thailand	Cross‐sectional	Both healthcare workers and non‐ healthcare workers (≥ 18 years) (32.0 years (NR); 73.5%)	NA	805	NA	HD, classified as mixed (primarily ICD)^a^	An unidentified questionnaire (self‐reported)	6
Teo et al. [31] Malesia	Cross‐sectional	Healthcare workers (34.6 years (±7.5); 80.8%)	NA	1004	NA	HD, classified as mixed (primarily ICD)^a^	NOSQ‐2002 based questionnaire (self‐reported and dermatological examination)	6
Tesfaye et al. [35] Ethiopia	Cross‐sectional	Cleaners (private, public healthcare) (31.0 years (±7.9); 73.8%)	NA	408	NA	CD, classified as mixed (primarily ICD)^a^	NOSQ‐2002 questionnaire (interview)	8
Thetkathuek et al. [30] Thailand	Cross‐sectional	Fiberboard (MDF) production workers (39.9 years (±9.8); 66.6%)	NA	323	NA	CD, classified as mixed^a^	A NOSQ‐2002‐based questionnaire (self‐reported)	5

Abbreviation: IS, individual susceptibility.

^a^ Cross‐sectional studies reporting both irritant and allergen exposures were classified as mixed (irritant contact dermatitis (ICD) + allergic contact dermatitis (ACD)); predominant dermatitis type was indicated per study.

**TABLE 2:** Overview of the investigated work‐related exposures, non‐work and work‐related factors in adjustment, and their impact on risk estimates.

Exposure category	References	Work‐related exposure	Non‐work and work‐related factors in adjustment		Univariate analysis (95% CI)^a^	Multivariate analysis (95% CI)^b^	Percentage change in risk estimate (%)
			Non‐work‐related factors	Work‐related factors			
Wet work	Ahn et al. [23]	Wet work (contact with wastewater)	Sex, age (15–44 years old vs. ≥ 45 years old), commune (location); previous history of skin problems, seasonal conditions (wet vs. dry), wet work exposure at home	Use of personal protective measures	OR 4.70 (1.90–11.61)	OR 3.00 (1.13–7.94)	▼ 36.2
	Brands et al._AD1 [36]	Occupational wet work exposure^(1)^	Sex, age, AD, contact allergy, wet work at home	Wet work	OR 1.73 (1.61–1.86)	OR 1.36 (1.25–1.48)	▼ 21.4
		Direct contact with water, fluids and/or moist products at work (hours/day)^(2)^			0.5‐1 h/day: OR 1.32 (1.21–1.44) 1‐2 h/day: OR 1.37 (1.23–1.53) > 2 h/day: OR 1.93 (1.74–2.15)	0.5‐1 h/day: OR 1.12 (1.02–1.24) 1‐2 h/day: OR 1.09 (0.96–1.23) > 2 h/day: OR 1.48 (1.31–1.67)	▼ 15.2 ▼ 20.4 ▼ 23.3
		Hand washing frequency (times/day)^(3)^			5–10 times/day: OR 1.30 (1.21–1.41) 10–20 times/day: OR 1.71 (1.55–1.88) > 20 times/day: OR 2.20 (1.95–2.48)	5–10 times/day: OR 1.09 (1.01–1.19) 10–20 times/day: OR 1.23 (1.10–1.37) > 20 times/day: OR 1.54 (1.35–1.76)	▼ 16.2 ▼ 28.1 ▼ 30.0
	Brands et al._AD2 [36]	Occupational wet work exposure^(1)^	Sex, age, AD, contact allergy, wet work at home, educational attainment, income	Wet work, employment status, work hours	OR 1.73 (1.61–1.87)	OR 1.35 (1.22–1.49)	▼ 22.0
		Direct contact with water, fluids and/or moist products at work (hours/day)^(2)^			0.5‐1 h/day: OR 1.32 (1.21–1.44) 1‐2 h/day: OR 1.37 (1.23–1.53) > 2 h/day: OR 1.93 (1.74–2.15)	0.5‐1 h/day: OR 1.14 (1.02–1.27) 1‐2 h/day: OR 1.06 (0.92–1.23) > 2 h/day: OR 1.52 (1.31–1.76)	▼ 13.6 ▼ 22.6 ▼ 21.2
		Hand washing frequency (times/day)^(3)^			5–10 times/day: OR 1.30 (1.21–1.41) 10–20 times/day: OR 1.71 (1.55–1.88) > 20 times/day: OR 2.20 (1.95–2.48)	5–10 times/day: OR 1.16 (1.05–1.27) 10–20 times/day: OR 1.29 (1.14–1.47) > 20 times/day: OR 1.54 (1.32–1.80)	▼ 10.8 ▼ 24.6 ▼ 30.0

	Hamnerius et al. 2021 [38]	Frequency of using soap (times/day)	Sex, age, AD, wet work exposure at home, non‐occupational face mask exposure	Working hours	11–20 times/day: OR 1.09 (0.95–1.25) > 20 times/day: OR 1.52 (1.30–1.78)	11–20 times/day: OR 1.16 (0.97–1.37) > 20 times/day: OR 1.78 (1.43–2.21)	▼ 6.4 ▲ 17.1
	Hamnerius et al. 2018_AD1 [37]	Frequency of hand washing with soap at work (times/day)	Sex, age	NA	11–20 times/day: OR 1.14 (1.01–1.29) > 20 times/day: OR 1.22 (1.07–1.39)	11–20 times/day: OR 1.25 (1.06–1.46) > 20 times/day: OR 1.25 (1.05–1.49)	▲ 9.6 ▲ 2.5
	Hamnerius et al. 2018_AD2 [37]		Sex, age, wet work exposure at home	NA	11–20 times/day: OR 1.14 (1.01–1.29) > 20 times/day: OR 1.22 (1.07–1.39)	11–20 times/day: OR 1.27 (1.05–1.53) > 20 times/day: OR 1.31 (1.06–1.62)	▲ 11.4 ▲ 7.4
	Hamnerius et al. 2018_AD3 [37]		Sex, age, wet work exposure at home, BMI, daily smoking, stress	NA	11–20 times/day: OR 1.14 (1.01–1.29) > 20 times/day: OR 1.22 (1.07–1.39)	11–20 times/day: OR 1.28 (1.06–1.55) > 20 times/day: OR 1.30 (1.04–1.62)	▲ 12.3 ▲ 6.6
	Hamnerius et al. 2018_AD4 [37]		Sex, age, wet work exposure at home, BMI, daily smoking, stress, history of AD	NA	11–20 times/day: OR 1.14 (1.01–1.29) > 20 times/day: OR 1.22 (1.07–1.39)	11–20 times/day: OR 1.33 (1.08–1.64) > 20 times/day: OR 1.43 (1.12–1.83)	▲ 16.7 ▲ 17.2
	Huang et al. [25]	Hand washing frequency (times/day)	Sex, caring for children under 4 years old, food allergy, allergic conjunctivitis	Alcoholic hand disinfectant use, frequent use of hair dye	10–20 times/day: OR 0.61 (0.28–1.34) 20–50 times/day: OR 1.15 (0.46–2.89) > 50 times/day: OR 4.76 (0.93–24.33)	10–20 times/day: OR 0.54 (0.25–1.18) 20–50 times/day: OR 1.13 (0.47–2.73) > 50 times/day: OR 4.86 (1.04–22.76)	▼ 11.5 ▼ 1.7 ▲ 2.1
Lee et al. [26]	Hand washing frequency (times/day)	Age, AD	Department, work duration, night duty, glove wearing time, hand moisturiser	10–19 times/day: OR 1.22 (0.72–2.07) 20–29 times/day: OR 5.17 (2.44–10.97) > 30 times/day: OR 12.18 (3.51–42.29)	10–19 times/day: OR 1.31 (0.72–2.36) 20–29 times/day: OR 5.77 (2.53–13.17) > 30 times/day: OR 13.08 (3.48–49.16)	▲ 7.4 ▲ 11.6 ▲ 7.4

	Lund et al._AD1 [41]	Probability of having wet hands ≥ 2 h/day	Sex, age, resident children age of ≤ 4	NA	> 25%–50%: OR 1.94 (1.76–2.13) > 50%–75%: OR 2.04 (1.87–2.22) > 75%: OR 2.96 (2.59–3.38)	> 25%–50%: OR 1.66 (1.50–1.83) > 50%–75%: OR 2.62 (2.40–2.87) > 75%: OR 3.81 (3.32–4.37)	▲ 14.4 ▲ 28.4 ▲ 28.7
	Lund et al._AD2 [41]		Sex, age, educational level, income level, resident children age of ≤ 4, residence, AD, positive patch test, the estimate of smoking	NA	> 25%–50%: OR 1.94 (1.76–2.13) > 50%–75%: OR 2.04 (1.87–2.22) > 75%: OR 2.96 (2.59–3.38)	> 25%–50%: OR 1.44 (1.30–1.60) > 50%–75%: OR 2.27 (2.07–2.49) > 75%: OR 2.97 (2.57–3.43)	▼ 25.8 ▲ 11.3 ▲ 0.3
Mekonnen et al. [33]	Hand washing frequency (times/day)	Educational level, monthly salary in Ethiopian birr, personal history of allergy	Work experience, working hours per day, job satisfaction, pairs of hand gloves used per day, periodic medical examination, occupational safety and health (training, hours of hand gloves used per day)	6–10 times/day: OR 1.14 (0.66–1.98) > 10 times/day: OR 9.86 (5.60–17.35)	6–10 times/day: OR 1.11 (0.85–1.22) > 10 times/day: OR 1.80 (1.06–3.07)	▼ 2.6 ▼ 81.7
Wet work	Minamoto et al. [27]	Hand washing frequency (times/day)	Sex, atopic dermatitis, asthma, allergic rhinitis/conjunctivitis, dry skin, metal allergy	Profession, work hours per week, years working in dentistry, duration of glove wearing, use of alcohol‐based hand‐scrub disinfectants	≥ 11 times/day: OR 1.30 (0.89–1.89)	≥ 11 times/day: OR 1.60 (0.99–2.58)	▲ 23.1
	Smith et al. [28]	Hand washing frequency (times/shift)	Age, alcohol consumption, presence of systemic allergic disease	Latex glove usage, total duration of employment, working in the surgery department	> 15 times/shift: OR 2.10 (1.30–3.40)	> 15 times/shift: OR 2.00 (1.19–3.37)	▼ 4.8
	Techasatian et al. [29]	Hand washing frequency (times/day)	Sex, previous hand eczema, underlying skin disease (atopic dermatitis), wearing gloves in everyday life	Occupation, handwashing methods during COVID‐19 pandemic	≥ 10 times/day: OR 1.55 (1.09–2.21)	≥ 10 times/day: OR 1.70 (1.05–2.75)	▲ 9.7

	Teo et al. [31]	Hand washing frequency (times/day)	Atopic eczema, allergic rhinitis, taking care of children under 4 years old	Use of alcoholic disinfectants, use of moisturiser hand cream	6–10 times/day: OR 1.62 (0.94–2.79) 11–20 times/day: OR 2.02 (1.17–3.50) ≥ 20 times/day: OR 3.19 (1.82–5.59)	6–10 times/day: OR 2.43 (1.31–4.50) 11–20 times/day: OR 3.34 (1.76–6.34) ≥ 20 times/day: OR 5.61 (2.88–10.94)	▲ 50.0 ▲ 65.3 ▲ 75.9
Tesfaye et al. [35]	Hand washing frequency (times/day)	Sex, personal history of eczema	Frequency of hand sanitising, periodic medical examination, information on workplace health and safety, training of skin hazards, job satisfaction	11–20 times/day: OR 1.11 (0.66–1.88) > 20 times/day: OR 1.93 (1.22–3.05)	11–20 times/day: OR 1.07 (0.60–1.92) > 20 times/day: OR 1.73 (1.03–2.91)	▼ 3.6 ▼ 10.4
Glove use	Brands et al._AD1 [36]	Glove wear time (hours/day)	Sex, age, AD, contact allergy, wet work at home	Wet work	0.5‐1 h/day: OR 1.83 (1.60–2.09) 1‐2 h/day: OR 1.72 (1.47–2.00) > 2 h/day: OR 1.92 (1.70–2.17)	0.5–1 h/day: OR 1.48 (1.28–1.72) 1–2 h/day: OR 1.36 (1.15–1.61) > 2 h/day: OR 1.48 (1.29–1.69)	▼ 19.1 ▼ 20.9 ▼ 22.9
	Brands et al._AD2 [36]		Sex, age, AD, contact allergy, wet work at home, educational attainment, income	Wet work, employment status, work hours	0.5‐1 h/day: OR 1.83 (1.60–2.09) 1‐2 h/day: OR 1.72 (1.47–2.00) > 2 h/day: OR 1.92 (1.70–2.17)	0.5–1 h/day: OR 1.42 (1.19–1.68) 1–2 h/day: OR 1.27 (1.05–1.55) > 2 h/day: OR 1.53 (1.31–1.78)	▼ 22.4 ▼ 26.2 ▼ 20.3
	Hamnerius et al. 2021 [38]	Glove wear time (hours/day)^(1)^	Sex, age, AD, wet work exposure at home, non‐occupational face mask exposure	Working hours	1‐3 h/day: OR 1.27 (1.09–1.48) > 3 h/day: OR 1.59 (1.36–1.85)	1‐3 h/day: OR 1.18 (0.99–1.40) > 3 h/day: OR 1.33 (1.10–1.61)	▼ 7.1 ▼ 16.4
	Hamnerius et al. 2021 [38]	Pairs of hand gloves used (number of pairs/day)^(2)^	Sex, age, AD, wet work exposure at home, non‐occupational face mask exposure	Working hours	11–20 pairs/day: OR 1.13 (0.96–1.34) > 20 pairs/day: OR 1.41 (1.22–1.63)	11–20 pairs/day: OR 0.97 (0.79–1.18) > 20 pairs/day: OR 1.03 (0.84–1.26)	▼ 14.2 ▼ 27.0
	Hamnerius et al. 2018_AD1 [37]	Glove wear time (hours/day)	Sex, age	NA	1‐3 h/day: OR 1.29 (1.10–1.52) > 3 h/day: OR 1.60 (1.35–1.90)	1‐3 h/day: OR 1.21 (1.02–1.44) > 3 h/day: OR 1.50 (1.24–1.81)	▼ 6.2 ▼ 6.3

	Hamnerius et al. 2018_AD2 [37]		Sex, age, wet work exposure at home	NA	1‐3 h/day: OR 1.29 (1.10–1.52) > 3 h/day: OR 1.60 (1.35–1.90)	1‐3 h/day: OR 1.15 (0.94–1.40) > 3 h/day: OR 1.42 (1.14–1.77)	▼ 10.9 ▼ 11.3
	Hamnerius et al. 2018_AD3 [37]		Sex, age, wet work exposure at home, BMI, daily smoking, stress	NA	1‐3 h/day: OR 1.29 (1.10–1.52) > 3 h/day: OR 1.60 (1.35–1.90)	1‐3 h/day: OR 1.17 (0.95–1.43) > 3 h/day: OR 1.42 (1.13–1.78)	▼ 9.3 ▼ 11.3
	Hamnerius et al. 2018_AD4 [37]		Sex, age, wet work exposure at home, BMI, daily smoking, stress, history of AD	NA	1‐3 h/day: OR 1.29 (1.10–1.52) > 3 h/day: OR 1.60 (1.35–1.90)	1‐3 h/day: OR 1.20 (0.97–1.50) > 3 h/day: OR 1.47 (1.14–1.89)	▼ 7.0 ▼ 8.1
	Huang et al. [25]	Glove wear time (hours/day)	Sex, caring children under 4 years old, food allergy, allergic conjunctivitis	Hand washing frequency, alcoholic hand disinfectant use, frequent use of hair dye	1‐4 h/day: OR 1.26 (0.61–2.63) > 4 h/day: OR 1.59 (0.69–3.65)	1‐4 h/day: OR 1.27 (0.64–2.55) > 4 h/day: OR 1.47 (0.66–3.28)	▲ 0.8 ▼ 7.5
	Lee et al. [26]	Glove wear time (min/day)	Age, AD	Department, periods of employment, night duty, hand washing frequency	1–5 min: OR 1.55 (1.00–2.42) > 5 min: OR 2.02 (1.12–3.65)	1–5 min: OR 1.60 (0.96–2.65) > 5 min: OR 1.99 (1.01–3.92)	▲ 3.2 ▼ 1.5
	Lund et al._AD1 [41]	Probability of wearing gloves at work ≥ 2 h/day	Sex, age, resident children age of ≤ 4, residence	NA	> 25%–50%: OR 2.39 (2.15–2.66) > 50%–75%: OR 2.09 (1.88–2.34) > 75%: OR 2.22 (1.97–2.51)	> 25%–50%: OR 2.10 (1.88–2.35) > 50%–75%: OR 2.64 (2.35–2.95) > 75%: OR 2.75 (2.43–3.11)	▼ 12.1 ▲ 26.3 ▲ 23.9
Lund et al._AD2 [41]		Sex, age, educational level, income level, resident children age of ≤ 4, residence, AD, positive patch test, likelihood of smoking	NA	> 25%–50%: OR 2.39 (2.15–2.66) > 50%–75%: OR 2.09 (1.88–2.34) > 75%: OR 2.22 (1.97–2.51)	> 25%–50%: OR 1.72 (1.53–1.93) > 50%–75%: OR 2.23 (1.99–2.50) > 75%: OR 2.50 (2.20–2.85)	▼ 28.0 ▲ 6.7 ▲ 12.6

Mekonnen et al. [33]	Glove wear time (hours/day)^(1)^	Educational level, monthly salary in Ethiopian birr, personal history of allergy	Work experience, working hours per day, hand washing frequency per day, job satisfaction, pairs of hand gloves used per day, periodic medical examination, occupational safety and health training	2‐6 h/day: OR 1.03 (0.31–2.11) > 6 h/day: OR 1.70 (0.40–7.22)	2‐6 h/day: OR 1.01 (0.15–1.93) > 6 h/day: OR 1.51 (0.58–3.94)	▼ 1.9 ▼ 11.2
Glove use	Mekonnen et al. [33]	Pairs of hand gloves used (number of pairs/day)^(2)^	Educational level, monthly salary in Ethiopian birr, personal history of allergy	Work experience, working hours, hand washing frequency per day, job satisfaction, periodic medical examination, occupational safety and health training, hours of hand gloves used per day	1–5 pairs/day: OR 1.73 (0.92–3.28) > 5 pairs/day: OR 40.0 (17.98–89.00)	1–5 pairs/day: OR 1.53 (0.43–3.12) > 5 pairs/day: OR 3.22 (1.90–5.45)	▼ 11.6 ▼ 92.0
	Minamoto et al. [27]	Glove wear time (hours/day)	Sex, atopic dermatitis, asthma, allergic rhinitis/conjunctivitis, dry skin, metal allergy	Profession, work hours per week, years working in dentistry, times washing your hands, use of alcohol‐based hand‐scrub disinfectants	> 6 h/day: OR 1.40 (0.99–1.98)	> 6 h/day: OR 1.20 (0.75–1.92)	▼ 14.3
	Hamnerius et al. 2021 [38]	Alcoholic hand disinfectants frequency (times/day)	Sex, age, AD, wet work exposure at home, non‐occupational face mask exposure	Working hours	21–50 times/day: OR 1.23 (1.00–1.50) > 50 times/day: OR 1.79 (1.47–2.18)	21–50 times/day: OR 1.00 (0.79–1.26) > 50 times/day: OR 1.03 (0.80–1.33)	▼ 18.7 ▼ 42.5
Hand hygiene and sanitization practices	Hamnerius et al. 2018_AD1 [37]	Alcoholic hand disinfectants frequency (times/day)	Sex, age	NA	21–50 times/day: OR 1.10 (0.94–1.29) > 50 times/day: OR 1.27 (1.09–1.48)	21–50 times/day: OR 0.96 (0.76–1.20) > 50 times/day: OR 0.92 (0.73–1.16)	▼ 12.7 ▼ 27.6
	Hamnerius et al. 2018_AD2 [37]		Sex, age, wet work exposure at home	NA	21–50 times/day: OR 1.10 (0.94–1.29) > 50 times/day: OR 1.27 (1.07–1.48)	21–50 times/day: OR 0.95 (0.73–1.23) > 50 times/day: OR 0.95 (0.72–1.25)	▼ 13.6 ▼ 25.2

	Hamnerius et al. 2018_AD3 [37]		Sex, age, wet work exposure at home, BMI, daily smoking, stress	NA	21–50 times/day: OR 1.10 (0.94–1.29) > 50 times/day: OR 1.27 (1.07–1.48)	21–50 times/day: OR 0.90 (0.69–1.18) > 50 times/day: OR 0.91 (0.68–1.21)	▼ 18.2 ▼ 28.3
	Hamnerius et al. 2018_AD4 [37]		Sex, age, wet work exposure at home, BMI, daily smoking, stress, history of AD	NA	21–50 times/day: OR 1.10 (0.94–1.29) > 50 times/day: OR 1.27 (1.07–1.48)	21–50 times/day: OR 0.82 (0.61–1.09) > 50 times/day: OR 0.76 (0.56–1.03)	▼ 25.5 ▼ 40.2
	Huang et al. [25]	Alcoholic hand disinfectants frequency (times/day)	Sex, caring children under 4 years old (hours daily), food allergy, allergic conjunctivitis	hand washing (times daily), frequent use of hair dye	5–10 times/day: OR 0.69 (0.29–1.62) > 10 times/day: OR 0.43 (0.17–1.09)	5–10 times/day: OR 0.75 (0.33–1.69) > 10 times/day: OR 0.48 (0.19–1.20)	▲ 8.7 ▲ 11.6
	Minamoto et al. [27]	Alcoholic hand disinfectants use (no)	Sex, atopic dermatitis, asthma, allergic rhinitis/conjunctivitis, dry skin, metal allergy	Professions, work hours per week, years working in dentistry, times washing your hands, duration of glove wearing	OR 1.50 (1.01–2.22)	OR 1.40 (0.90–2.19)	▼ 6.7
	Teo et al. [31]	Alcoholic hand disinfectants frequency (times/day)	Atopic eczema, allergic rhinitis, taking care of children under 4 years old	Hand washing frequency, use of moisturiser hand cream	6–10 times/day: OR 0.53 (0.38–0.76) 11–20 times/day: OR 0.66 (0.46–0.97) ≥ 20 times/day: OR 0.75 (0.51–1.10)	6–10 times/day: OR 0.53 (0.30–0.79) 11–20 times/day: OR 0.56 (0.37–0.87) ≥ 20 times/day: OR 0.49 (0.31–0.78)	— ▼ 15.2 ▼ 34.7
Tesfaye et al. [35]	Hand sanitising frequency (times/day)	Sex, personal history of eczema	Frequency of hand washing, periodic medical examination, information on workplace health and safety, training of skin hazards, job satisfaction	6–10 times/day: OR 0.82 (0.46–1.48) 11–20 times/day: OR 0.76 (0.44–1.29) > times/day: OR 0.72 (0.41–1.26)	6–10 times/day: OR 0.95 (0.51–1.76) 11–20 times/day: OR 0.82 (0.47–1.44) > 20 times/day: OR 0.75 (0.42–1.34)	▲ 15.9 ▲ 7.9 ▲ 4.2
Work duration and experience	Attwa et al. [32]	Work experience (> 10 years)	History of eczema, family history of atopy	Occupation, duration of work	OR 3.81 (1.06–15.35)	OR 2.38 (1.02–5.56)	▼ 37.5
	Brands et al._AD1 [36]	Work hours (hours/week)	Sex, age, AD, contact allergy, wet work at home	Wet work	9–16 h/week: OR 1.22 (1.03–1.45) 17–24 h/week: OR 1.20 (1.02–1.40) 25–32 h/week: OR 1.18 (1.01–1.38) 33–40 h/week: OR 0.92 (0.79–1.06) > 40 h/week: OR 0.88 (0.74–1.04)	9–16 h/week: OR 1.10 (0.90–1.33) 17–24 h/week: OR 1.06 (0.89–1.27) 25–32 h/week: OR 1.08 (0.91–1.29) 33–40 h/week: OR 1.04 (0.87–1.24) > 40 h/week: OR 1.08 (0.88–1.32)	▼ 9.8 ▼ 11.7 ▼ 8.5 ▲ 13.0 ▲ 22.7
	Brands et al._AD2 [36]		Sex, age, AD, contact allergy, wet work at home, educational attainment, income	Wet work, employment status, work hours	9–16 h/week: OR 1.22 (1.03–1.45) 17–24 h/week: OR 1.20 (1.02–1.40) 25–32 h/week: OR 1.18 (1.01–1.38) 33–40 h/week: OR 0.92 (0.79–1.06) > 40 h/week: OR 0.88 (0.74–1.04)	9–16 h/week: OR 1.09 (0.87–1.37) 17–24 h/week: OR 1.06 (0.85–1.33) 25–32 h/week: OR 1.08 (0.86–1.34) 33–40 h/week: OR 1.00 (0.80–1.24) > 40 h/week: OR 1.08 (0.84–1.38)	▼ 10.7 ▼ 11.7 ▼ 8.5 ▲ 8.7 ▲ 22.7
	Hajaghazadeh et al. [24]	Work experience (≤ 10 years)	Age, smoking	NR	OR 3.27 (2.08–6.67)	OR 3.14 (1.36–6.04)	▼ 4.0
	Lee et al. [26]	Work experience (years)	Age, AD	Department, night duty, hand washing frequency, glove wearing time, hand moisturiser	5–10 years: OR 1.30 (0.77–2.19) > 10 years: OR 0.61 (0.39–0.97)	5–10 years: OR 1.72 (0.90–3.29) > 10 years: OR 2.17 (0.79–5.94)	▲ 32.3 ▲ 255.7
Work duration and experience	Mekonnen et al. [33]	Work experience (years)	Educational level, monthly salary in Ethiopian birr, personal history of allergy	Working hours per day, hand washing frequency per day, job satisfaction, periodic medical examination, pairs of hand gloves used per day, occupational safety and health training, hours of hand gloves used per day	5–10 years: OR 1.41 (0.82–2.40) > 10 years: OR 2.82 (1.73–4.58)	5–10 years: OR 1.40 (0.02–2.19) > 10 years: OR 1.40 (0.04–1.59)	▼ 0.7 ▼ 50.4
		Working > 8 h/day	Educational level, monthly salary in Ethiopian birr, personal history of allergy	Work experience, job satisfaction, periodic medical examination, hand washing frequency per day, pairs of hand gloves used per day, occupational safety and health training, hours of hand gloves used per day	OR 7.37 (4.41–12.32)	3.80 (0.11–4.82)	▼ 48.4
	Minamoto et al. [27]	Working ≥ 40 h/week	Sex, atopic dermatitis, asthma, allergic rhinitis/conjunctivitis, dry skin, metal allergy	Professions, years working in dentistry, times washing your hands, duration of glove wearing, use of alcohol‐based hand‐scrub disinfectants	OR 1.30 (0.90–2.00)	OR 1.20 (0.70–1.90)	▼ 7.7
		Work experience in dentistry (year)	Sex, atopic dermatitis, asthma, allergic rhinitis/conjunctivitis, dry skin, metal allergy	Professions, work hours per week, times washing your hands, duration of glove wearing, use of alcohol‐based hand‐scrub disinfectants	≤ 10 years: OR 2.90 (1.80–4.60) 10.1–20.0 years: OR 2.10 (1.30–3.60)	≤ 10 years: OR 2.0 (1.20–3.50) 10.1–20.0 years: OR 1.70 (1.00–3.10)	▼ 31.0 ▼ 19.0

	Spee et al. [42]	Work experience with epoxy (per 10‐year increase)	Age	Number of working hours per week, ever having had an unusually high exposure to epoxy product during work, wearing no gloves other than chemically resistant gloves, wearing short sleeves, using skin cream	OR: 0.52 (0.39–0.69)	OR 0.41 (0.27–0.61)	▼ 21.2
		Working hours a week (per 10‐h increase)	Age	Years of working experience, ever having had an unusually high exposure to epoxy product during work, wearing no gloves other than chemically resistant gloves, wearing short sleeves, using skin cream	OR 1.89 (1.56–2.29)	OR 1.72 (1.39–2.14)	▼ 9.0
	Tamene et al. [34]	Employment pattern	Age	Work activity, work hours per week, use of personal protective clothing, pre‐employment safety training, knowledge of pesticide use and storage	Contractual: OR 1.73 (0.99–3.01) Permanent: OR 3.28 (1.69–6.36)	Contractual: OR 0.89 (0.39–2.03) Permanent: OR 1.76 (0.80–3.89)	▼ 48.6 ▼ 46.3
Working > 48 h/week		Age	Work activity, employment pattern, use of personal protective clothing, pre‐employment safety training, knowledge of pesticide use and storage	OR 1.61 (1.03–2.52)	OR 1.30 (0.80–2.12)	▼ 19.3
Occupational sectors and departments	Alluhayyan et al. [22]	Occupation‐based exposure (other allied practitioners*)	Sex, age	NA	Doctors: OR 1.36 (0.55–3.33) Nurses: OR 1.41 (0.64–3.13) Pharmacists: OR 2.64 (0.95–7.33)	Doctors: OR 1.93 (0.67–5.56) Nurses: OR 1.20 (0.45–3.22) Pharmacists: OR 3.69 (1.15–11.81)	▲ 41.9 ▼ 14.9 ▲ 39.8
	Lee et al. [26]	Department‐based exposure (outpatient department*)	Age, atopic dermatitis, hand moisturiser	Night duty, hand washing frequency, glove wearing time, hand moisturiser	Special unit: OR 1.81 (0.97–3.39) Regular ward: OR 2.23 (1.20–4.13)	Special unit: OR 1.06 (0.48–2.37) Regular ward: OR 1.36 (0.63–2.95)	▼ 41.4 ▼ 39.0
	Meding et al. [39] **	Occupation of dental technician	Sex	NA	Male: IRR 3.60 (2.30–5.60) Female: IRR 2.40 (1.70–3.30)		
	Minamoto et al. [27]	Occupation‐based exposure (dentist*)	Sex, atopic dermatitis, asthma, allergic rhinitis/conjunctivitis, dry skin, metal allergy	Work hours per week, years working in dentistry, times washing your hands, duration of glove wearing, use of alcohol‐based hand‐scrub disinfectants	Hygienists: OR 2.40 (1.40–4.10) Technician: OR 1.50 (0.50–4.20) Assistant: OR 2.10 (1.10–4.20) Receptionist: OR 2.00 (1.00–4.20)	Hygienists: OR 1.90 (0.70–5.00) Technician: OR 1.60 (0.50–4.90) Assistant: OR 2.10 (0.70–6.10) Receptionist: OR 2.00 (0.60–6.20)	▼ 20.8 ▲ 6.7 — —
	Smith et al. [28]	Department‐based exposure (other departments*)	Age, alcohol consumption, presence of systemic allergic disease	Latex glove usage, total duration of employment, number of hand washes	Surgery: OR 1.80 (1.10–3.20)	Surgery: OR 1.80 (1.00–3.20)	—
	Tamene et al. [34]	Type of farm labour (irrigation technician*)	Age	Employment pattern, work hours per week, use of personal protective clothing, pre‐employment safety training, knowledge of pesticide use and storage	Field worker: OR 1.28 (0.46–3.53) Bundle binder: OR 2.91 (1.01–8.41) Pesticide applicator: OR 0.86 (0.28–2.59)	Field worker: OR 1.78 (0.57–5.54) Bundle binder: OR 5.74 (2.12–15.55) Pesticide applicator: OR 1.06 (0.31–3.60)	▲ 39.1 ▲ 97.3 ▲ 23.3
Specific hazardous substances	Huang et al. [25]	Frequent use of hair dyes (yes)	Sex, caring children under 4 years old, food allergy, allergic conjunctivitis	Hand washing frequency, alcoholic hand disinfectant use	OR: 6.53 (1.55–27.58)	OR: 3.87 (1.11–13.55)	▼ 40.7
	Rönmark et al. [40]	Occupational exposure to gas, dust or fumes (GDF) (yes)	Sex, age, family history of asthma and rhinitis, raised on a farm, BMI, allergic sensitisation, number of siblings, childhood daycare	NA	OR: 1.73 (1.21–2.48)	OR: 2.08 (1.40–3.08)	▲ 20.2
	Spee et al. [42]	Ever having had an unusual high exposure to epoxy products	Age	Years of working experience, number of working hours per week, wearing no gloves other than chemically resistant gloves, wearing short sleeves, using skin cream	OR: 2.39 (1.28–4.46)	OR: 2.13 (1.01–4.51)	▼ 10.9
	Thetkathuek et al. [30]	Dust exposure	Metal allergy, exposure to bathroom cleaning chemicals, allergy of family members, exposure to bleach washing clothes, current smoking	NA	1–3 times/month: OR 2.50 (NR); p: 0.143 1–3 times/week: OR 3.30 (NR); p: 0.019 Everyday: OR 3.60 (NR); p: 0.004	1–3 times/month: OR 3.40 (0.70–16.90) 1–3 times/week: OR 4.90 (1.30–18.60) Everyday: OR 3.90 (1.60–9.60)	▲ 36.0 ▲ 48.5 ▲ 8.3

*Note*: Colour indicators: Red = increased risk, Green = protective effect, black = minimal change (< 10%); the **asterisk** (*) indicates the reference category; the **double asterisk** (**) indicates an effect modifier; Superscript numbers 1, 2 and 3 refer to distinct exposures reported within the same study. These correspond to the same numbered exposures shown under the same reference in Figures 3 and 4. The suffix AD(number) in the reference column (e.g., Brands et al._AD1) denotes distinct multivariate models within the same study, each adjusted for different combinations of covariates.

Abbreviation: AD, atopic dermatitis.

^a^ Unadjusted risk estimates reflect the impact of work‐related exposures on the likelihood of developing contact dermatitis (CD).

^b^ Adjusted risk estimates reflect the impact of work‐related exposures on the likelihood of developing contact dermatitis (CD), accounting for other variables.

### 3.3 Non‐Work Susceptibility Factors and Work‐Related Factors in Adjustment

A total of 44 distinct factors, including both non‐work susceptibility factors and work‐related factors, were adjusted for in the analyses of included studies. Twenty studies examined combinations of susceptibility factors, while one study specifically assessed the role of sex [39]. We identified five main categories of non‐work‐related susceptibility factors: demographic, socioeconomic, host‐related intrinsic, lifestyle, and exposures outside work. Detailed examples of these categories are presented in Figure 2. Additionally, 15 studies adjusted for work‐related factors beyond the main exposure, which were grouped into two categories. The first, exposures at work, included variables related to the work environment and job characteristics. The second, behavioural and preventive measures, encompassed factors associated with individual practices and protective strategies. A detailed overview of these factors is provided in Figure 2.

### 3.4 The Role of Non‐Work Susceptibility Factors and Work‐Related Factors on Exposure‐CD Associations

We evaluated whether the size and the direction of the univariable associations between work‐related exposures and CD changed when non‐work susceptibility factors and work‐related factors were adjusted for in multivariable analyses in the selected studies. None of the studies explicitly used the terms “confounder” or “effect modifier.” However, confounding appears to have been addressed by including various factors in multivariable analyses [22, 23, 24, 25, 26, 27, 28, 29, 30, 31, 32, 33, 34, 35, 36, 37, 38, 40, 41, 42]. Additionally, one study appeared to consider an effect modifier by presenting stratified analyses [39]. The number of studies per exposure category ranged from 4 to 13 studies, with some studies appearing in multiple exposure categories. The number of factors adjusted for in multivariable analyses ranged from 2 to 11 variables. Although many studies used statistical significance in univariable analyses to select variables for multivariate models, none provided a broader conceptual or theoretical rationale—such as confounding or biological plausibility—for their choices. These studies were not primarily designed to investigate the role of individual susceptibility factors, which may limit the interpretability of our findings.

**FIGURE 3:**
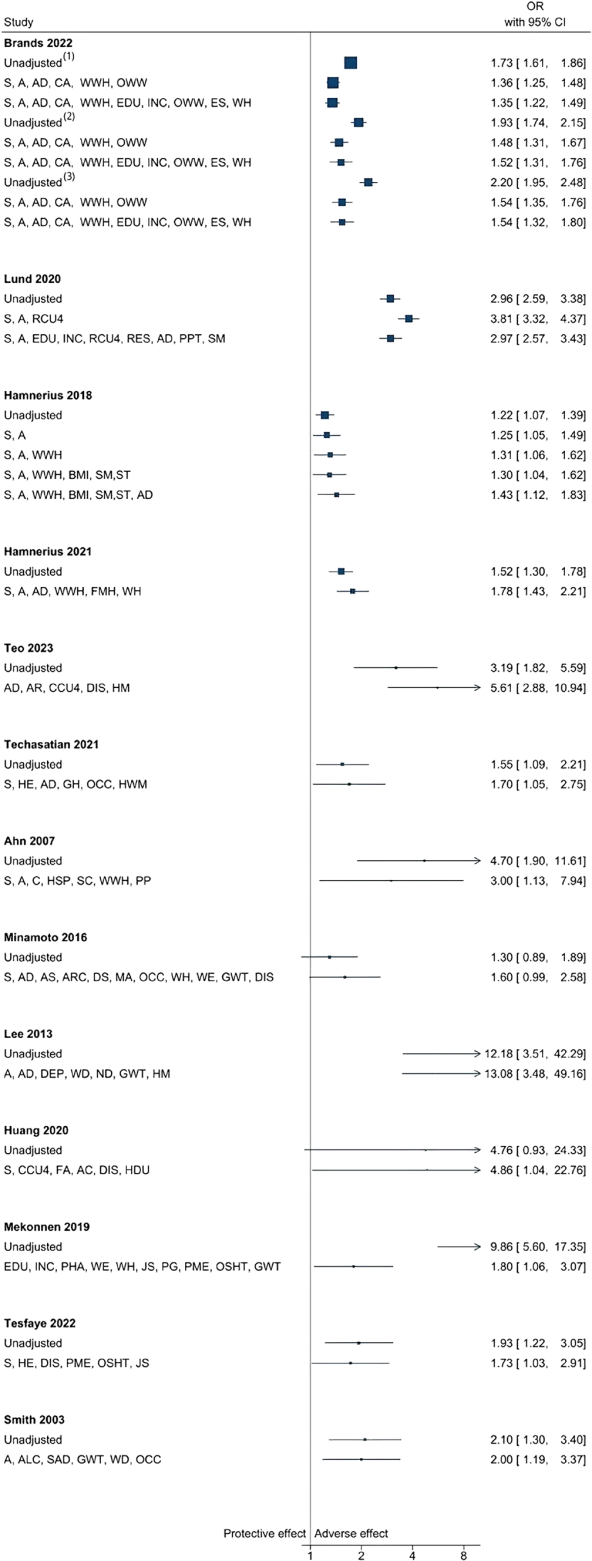
Forest plot of unadjusted and adjusted odds ratios for the exposure category “wet work (WW)” across studies. Superscript numbers 1, 2 and 3 refer to distinct exposures reported within the same study. These correspond to the specific exposures listed under the same reference in Table 2. S, sex; A, age; AD, (history of) atopic dermatitis/eczema; CA, contact allergy; WWH, wet work at home; OWW, occupational wet work; EDU, educational attainment; INC, income; ES, employment status; WH, working hours per day/week; RES, residence; RDC4, resident children under 4; PPT, positive patch test; SM, (estimate of) smoking; BMI, Body Mass Index; ST, stress; FMH, face mask use at home; AR, allergic rhinitis; CCU4, childcare for children under four; DIS, alcohol‐based hand scrub disinfectants (use/frequency); HM, hand moisturiser; GH, glove use at home; OCC, occupation; HWM, hand washing method; HSP, history of skin problems; SC, seasonal conditions; PP, personal protective measures use; AS, asthma; ARC, allergic rhinoconjunctivitis; DS, dry skin; MA, metal allergy; WE, work experience; GWT, glove wear time; DEP, work department; WD, work duration; ND, night duty; FA, food allergy; AC, allergic conjunctivitis; HDU, frequent hair dye use; PHA, personal history of allergy; JS, job satisfaction; PG, pairs of gloves used per day; PME, periodic medical examination; OSHT, occupational safety and health (OSH) training; ALC, alcohol consumption; SAD, systematic allergic diseases.

**FIGURE 4:**
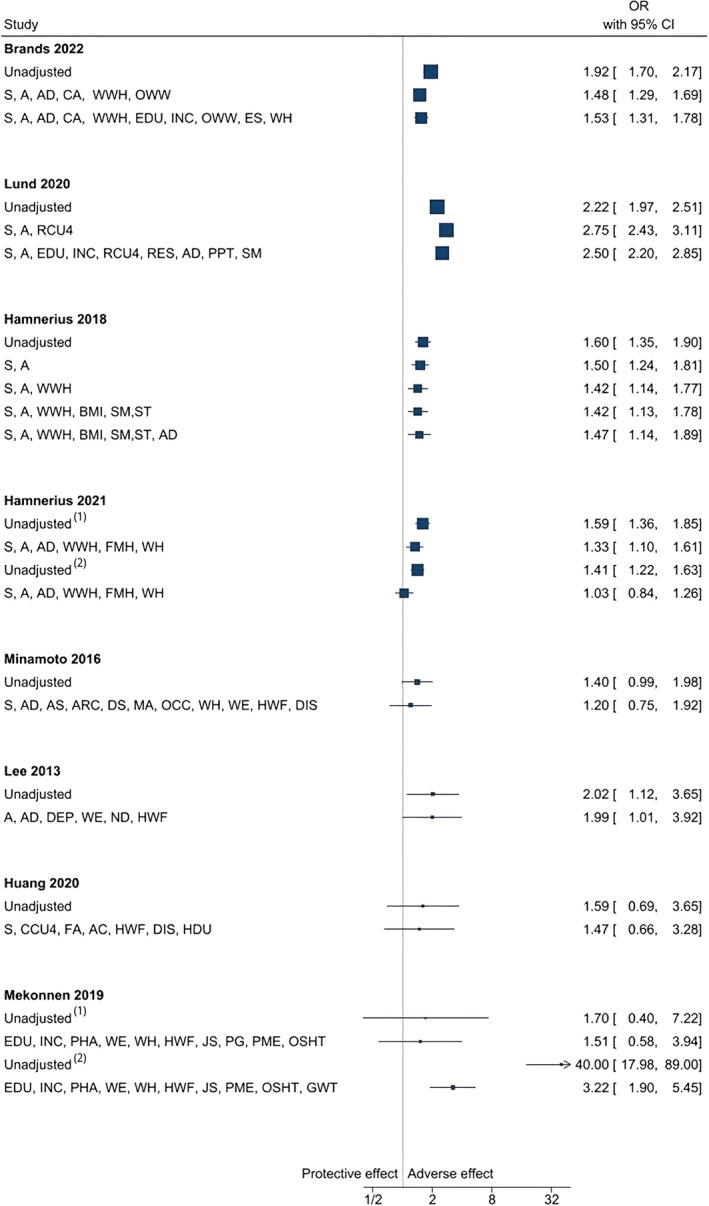
Forest plot of unadjusted and adjusted odds ratios for the exposure category “glove use” across studies. Superscript numbers 1 and 2 refer to distinct exposures reported within the same study. These correspond to the specific exposures listed under the same reference in Table 2. S, sex; A, age; AD, (history of) atopic dermatitis/eczema; CA, contact allergy; WWH, wet work at home; OWW, occupational wet work; EDU, educational attainment; INC, income; ES, employment status; WH, working hours per day/week; RDC4, resident children under 4; RES, residence; PPT, positive patch test; SM, (estimate of) smoking; BMI, Body Mass Index; ST, stress; FMH, face mask use at home; AS, asthma; ARC, allergic rhinoconjunctivitis; DS, dry skin; MA, metal allergy; OCC, occupation; WE, work experience; HWF, hand washing frequency; DIS, alcohol‐based hand scrub disinfectants (use/frequency); DEP, work department; ND, night duty; FA, food allergy; AC, allergic conjunctivitis; HDU, frequent hair dye use; PHA, personal history of allergy; JS, job satisfaction; PG, pairs of gloves used per day; PME, periodic medical examination; OSHT, occupational safety and health (OSH) training; GWT, glove wear time.

**FIGURE 5:**
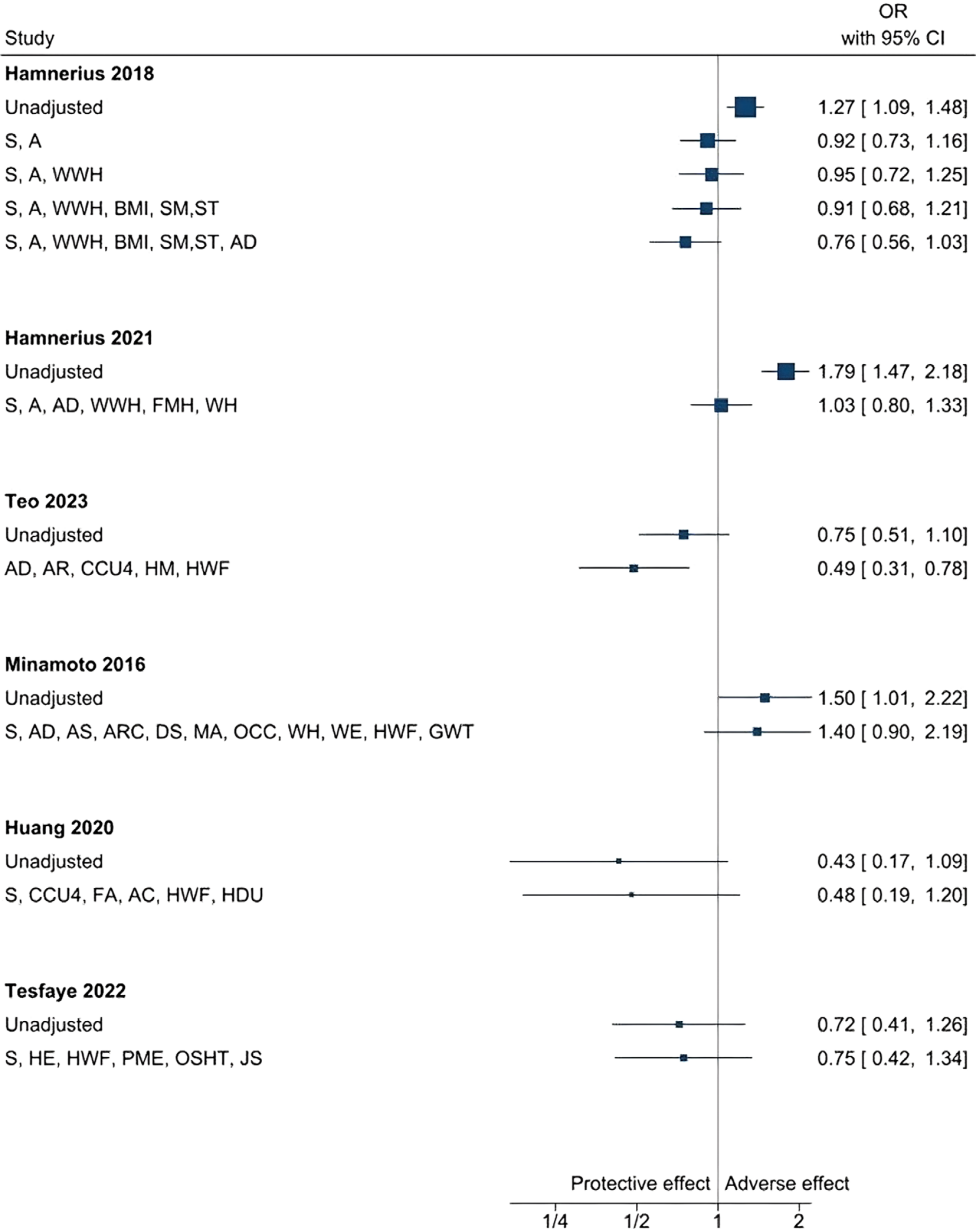
Forest plot of unadjusted and adjusted odds ratios for the exposure category “hand hygiene and sanitisation practices (HHS)” across studies. S, sex; A, age; WWH, wet work at home; BMI, Body Mass Index; SM, (estimate of) smoking; ST, stress; AD, (history with) atopic dermatitis/eczema; FMH, face mask use at home; WH, working hours per day/week; AR, allergic rhinitis; CCU4, children care under four; HM, hand moisturiser; HWF, hand washing frequency; AS, asthma; ARC, allergic rhinoconjunctivitis; DS, dry skin; MA, metal allergy; OCC, occupation; WE, work experience; GWT, glove wear time; FA, food allergy; AC, allergic conjunctivitis; HDU, frequent hair dye use; HE, (history with) hand eczema; PME, periodic medical examination; OSHT, occupational safety and health (OSH) training; JS, job satisfaction.

Looking at the differences between univariable (work exposures only) and multivariable analyses (additionally included non‐work susceptibility factors and work‐related factors), we observed considerable variations in the direction and magnitude of the role of non‐work susceptibility factors and additional adjustment for work‐related factors on the association between work‐related exposures and CD. The influence of adjusting for non‐work susceptibility factors and work‐related factors was examined separately for each exposure category outlined below:

- **Wet work** was the most frequently studied category, including handwashing frequency, prolonged wet hands (≥ 2 h/day), or soap use. Unadjusted ORs generally showed a positive association between wet work exposure and CD, with most studies reporting ORs above 1, ranging from 0.61 to 12.18, as illustrated in our forest plot (see Figure 3) [23, 25, 26, 27, 28, 29, 31, 33, 35, 36, 37, 38, 41]. This trend persisted after adjusting for non‐work susceptibility factors and work‐related factors, with ORs still generally above 1 and changes ranging from 0.3% to 82%. However, the direction of change after adjustment varied between studies, with most indicating a decrease in ORs [23, 25, 28, 33, 35, 36].

- **Glove use** was measured by wear time or number of glove pairs used daily. Wearing gloves was generally associated with an increased risk of CD in the univariable model, with ORs above 1 (ranging from 1.03 to 40.0) [25, 26, 27, 33, 36, 37, 38, 41]. In the multivariable model, the ORs remained above 1; however, the direction of the associations became inconsistent after adjusting for non‐work‐related susceptibility factors and work‐related exposures (see Figure 4). While the ORs predominantly decreased, some studies showed an increase in ORs in specific exposure categories, such as having a > 50% probability of wearing gloves at work for more than 2 h/day [41].

- **Hand hygiene and sanitisation practices**, particularly alcohol‐based disinfectant use, varied widely across studies—from fewer than 10 to over 50 uses per day. Univariable models showed considerable variability in ORs, with approximately half of the studies reporting ORs above 1 and others below 1, indicating inconsistent associations (see Figure 5). After adjusting for non‐work susceptibility factors and work‐related factors, most studies suggested a negative association, indicating a reduced likelihood of CD among individuals using alcohol‐based disinfectants at work [31, 37, 38], with three studies reporting risk reductions greater than 10% [31, 37, 38].

- **Work duration and experience**, measured by daily or weekly hours or years, showed that prolonged work experience (> 10 years) generally increased the risk of CD, with ORs ranging from 0.52 to 3.81 in the univariable models [24, 26, 27, 33, 42]. Univariable analysis showed inconsistent patterns for weekly working hours, with ORs ranging from 0.88 to 7.37 [27, 33, 34, 36, 42]. After adjustment, more than 10 years of work experience predominantly indicated a protective effect, reducing ORs by 1% to 50%, although the ORs mostly remained above 1. Notably, Lee et al. observed an increased risk among nursing staff with 5–10 years and more than 10 years of work experience after adjustment, with OR increases of 32% and 256% [26]. Adjusted ORs for working hours show mixed results. Brands et al. found a negative association for working 9–32 h/week (−8.5% to −11.7%), but a positive association for working > 33 h/week (+8.7% to +22.7%) [36]. In contrast, two studies reported protective effects for working ≥ 40 and > 48 h/week compared to working less than 40 and 48 h/week, with OR reductions of 8% and 19% [27, 34].
- **Occupational sector exposures** included multiple sectors with categorisation varying by profession, department, or work task. In healthcare, univariable analysis showed an increased CD risk in high‐exposure jobs compared to lower‐exposure jobs. Statistically significant associations were found for pharmacists (OR 2.64) [22], nurses (OR 1.41) [22], doctors (OR 1.36) [22], regular ward staff (OR 2.23) [26], and surgery department staff (OR 1.8) [28] compared to lower‐exposed allied practitioners and health staff from the outpatient department. After adjustment, the direction and strength of the association varied. In some studies, the ORs increased after adjustment, while in others, they decreased. Doctors (OR 1.93) [22] and pharmacists (OR 3.69) [22] showed increased CD risk compared to other allied practitioners [22]. The special unit and regular ward departments showed a decrease in OR by 41% and 39% [26], respectively, compared to other outpatient departments, as did nurses (−14.9%) and hygienists (−20.8%) [22, 27]. Among dental professionals, technicians exhibited the highest risk, with male dental technicians showing a significantly higher incidence rate ratio (IRR 3.6) compared to females (IRR 2.4), suggesting greater susceptibility among males [39].
- **Exposure to specific hazardous substances** such as hair dye, epoxy, and fumes was mostly reported in binary (i.e., yes/no) formats or rough frequency categories and linked to an increased CD risk. Notable univariable associations were found for hair dye (OR 6.53) [25] and epoxy (OR 2.39) [42]. Dust exposure showed a dose–response association, with everyday exposure (OR 3.6) having the highest risk [30]. After adjusting for non‐work‐related susceptibility factors and work‐related factors, two studies showed decreased odds, particularly for hair dye (−40.7%) [25] and epoxy (−10.9%) [42], while two others reported increased odds, such as for moderate dust exposure (+48.5%) [30] and occupational exposure to gas, dust, or fumes (+20.2%) [40].

## 4 Discussion

### 4.1 Summary of Evidence

This scoping review identified a wide range of non‐work‐related susceptibility factors that may influence the association between work‐related exposures and CD. Identified factors include demographic (e.g., sex, age, residence, number of siblings), socioeconomic (e.g., education, income), personal/family atopic conditions (e.g., general atopy, AD, hand eczema), lifestyle (e.g., smoking, alcohol consumption, BMI, stress levels), and exposures outside work (e.g., wet work, glove use, and seasonal conditions). Fifteen studies also adjusted for additional work‐related factors besides the main exposure, such as workplace exposures and behaviours related to exposure and prevention. After accounting for these factors, the association between the work‐related exposures and CD changed; however, their effect and direction on the association varied widely. There was also considerable variation in the type and number of factors adjusted for across studies.

### 4.2 Interpretation of the Findings

This scoping review demonstrates that associations between work‐related exposures and CD are influenced by adjustments for both non‐work‐related susceptibility factors and additional work‐related variables. Sex, age, and personal or family atopic conditions were among the most frequently adjusted variables. However, the heterogeneity among the studies did not allow for pooling effect estimates for these factors, highlighting the need for more (standardised) reporting in this area.

Our findings, while distinctive in terms of study objective (i.e., individual susceptibility), align with existing literature on CD risk factors such as sex, age, and atopy. ICD is more prevalent among women due to higher exposure to irritants in certain occupations and household activities [15, 43]. While experimental studies found no difference in irritant reactivity between sexes, structural differences in skin—such as thicker epidermis in males—may contribute to sex‐based susceptibility [39, 44, 45]. Interestingly, one study found that male dental technicians were more susceptible to CD than females [39]. Age also showed inconsistent associations with CD. Some studies reported a decreased risk among older individuals, possibly due to reduced skin sensitivity. Other studies found a higher risk among younger workers, potentially related to their lack of experience and limited use of protective equipment [15, 24, 43]. Additionally, epidemiological studies have shown that individuals with a history of AD are at increased risk of developing ICD, and potentially ACD, likely due to a compromised skin barrier that allows irritants and allergens to trigger immune and inflammatory responses [6, 46]. Other atopic conditions, such as asthma, allergic rhinitis, and food allergies, may also influence susceptibility through aberrant immune responses, although the direct role of these atopic conditions on CD is less clear [40].

The findings show substantial variation in how both non‐work‐related susceptibility factors and work‐related factors influenced the association between work‐related exposures and CD. In some studies, adjustment increased the observed risk, while in others, adjustment led to a protective effect. This variation highlights the complexity of studying the contribution of individual susceptibility factors to the association between work‐related exposures and CD. Differences in study design, population characteristics, exposure assessment, and adjustment strategies may contribute to these inconsistencies.

Furthermore, most studies primarily aimed to examine the association between irritant and/or allergic exposures and CD, or to develop prediction models designed to estimate an individual's risk or likelihood of developing CD based on relevant exposures and susceptibility factors. While adjusting for multiple factors is essential to avoid obscuring the association between exposure and outcome, it complicates the interpretation of each factor's specific contribution. Consequently, the studies included in this review support conclusions only about the combined effect of all adjusted variables, rather than the distinct impact of individual factors. Notably, most studies did not justify their selection of covariates in multivariable analyses, leaving it unclear whether important variables were deliberately excluded for valid reasons or whether unnecessary (over)adjustments were made. Moreover, none reported assessing potential correlations between adjustment factors prior to model inclusion, which may have introduced statistical biases and led to misinterpretation of associations related to the work‐related exposure(s) of interest [47]. Furthermore, we found that 20 studies presumably addressed confounding by adjusting for various factors, with only one addressing effect modification and none for mediation. This highlights the need for more studies on individual susceptibility, with a specific focus on providing a clear rationale for the inclusion of adjustment factors and determining their role in the association between work‐related exposures and CD.

### 4.3 Strengths and Limitations

A key addition of this review is its focus on how non‐work‐related susceptibility factors and work‐related factors influence the association between work‐related exposures and CD. To isolate these effects, we included only studies that reported both adjusted and unadjusted estimates. While this approach enhanced the precision of our analysis, it significantly reduced the number of eligible studies for our review.

The inclusion of mostly cross‐sectional studies presents some potential limitations, although it is not clear to what extent this affected the effect of susceptibility on the adjusted analyses. These studies allowed investigation of individual susceptibility factors, particularly for ACD, using patch tests and registry data. However, they are subject to selection and recall biases, as workers with severe CD might leave high‐risk occupations (i.e., healthy worker survivor bias), and these studies often relied on self‐reported data [27].

A major challenge of this scoping review was the limited number of studies specifying whether the type of CD was primarily irritant or allergic. Most studies were labelled as mixed CD, but uncertainty about the diagnosis was present. Some exposures include irritants and/or allergens at the same time, and it was often unclear whether individuals had ICD, ACD, or both [48]. The definitions and criteria for diagnosing mixed occupational CD are not standardised and need careful clinical (including allergen patch testing) and exposure assessment, which was largely lacking in the included studies. Additionally, the lack of separate analyses for the effects on ACD and ICD represents a limitation in the current evidence base.

Another limitation was the inconsistent definitions of work‐related exposures and individual susceptibility factors, as reported in other studies [6, 9, 43]. For example, “wet work” has been defined in various ways, from broad descriptions like “contact with wastewater” to specific thresholds such as “washing hands > 50 times daily” or “having wet hands for ≥ 2 h/day.” Diepgen et al. also observed similar variability for “atopy,” which limits comparability and generalizability [9].

### 4.4 Implications for Research and Practice

Prospective cohort or case‐control studies are needed to examine the moderating effect of susceptibility factors in the association between work‐related exposures and CD. Research should isolate the impact of individual susceptibility factors. Although evidence for the role of non‐work‐related susceptibility factors in the development of CD remains limited, it is advisable to consider susceptibility factors when evaluating the association between work‐related risk factors and CD. In particular, age, sex, history of AD and hand eczema, smoking status, and ethnicity may influence individual susceptibility to occupational CD, as these factors can affect both the integrity of the skin barrier and the immune response—key elements in the pathogenesis of CD. Identifying these factors as moderating or confounding factors in epidemiological studies may improve the accuracy of exposure‐risk estimates for CD. Harmonising and incorporating standardised susceptibility factors when examining the causal relationship between work‐related exposures and CD is crucial. Additionally, standardising definitions for work‐related exposures and CD diagnostics will enhance study comparability and improve diagnostic precision.

When developing programmes for the prevention, diagnosis, and treatment of occupational CD, it is advisable to consider non‐modifiable factors alongside modifiable factors like smoking status. Non‐modifiable factors help identify high‐risk individuals, while modifiable factors offer intervention opportunities, making them essential for effective prevention strategies.

## 5 Conclusion

In conclusion, while susceptibility factors are often adjusted for, their exact impact and direction on the association between work‐related exposures and CD remain inconclusive. Most studies account for various work‐ and non‐work‐related factors, complicating the isolation of their individual contributions. Despite limited evidence for non‐work‐related susceptibility factors in developing CD, factors such as age, sex, history of AD and hand eczema, smoking status, and ethnicity should be considered when studying the association between work‐related exposures and CD. Additionally, our review highlights the need for more research investigating the role of individual susceptibility factors and to standardise adjustment factors and achieve greater consensus to distinguish between ICD and ACD.

## Author Contributions

**Renate Juścikowski:** conceptualisation; writing – original draft; methodology; formal analysis; project administration. **Pieter Coenen:** conceptualisation; data curation; supervision. **Sanja Kezic:** conceptualisation; validation; data curation; supervision. **Damien M. McElvenny:** conceptualisation; data curation; supervision. **Faridi S. Jamaludin:** conceptualisation; methodology. **Henk F. van der Molen:** conceptualisation; funding acquisition; data curation; supervision. **Jan L. Hoving:** conceptualisation; data curation; supervision. All authors contributed to reviewing and editing the manuscript.

## Conflicts of Interest

The authors declare no conflicts of interest.

## Supporting information


**Data S1:** cod70030‐sup‐0001‐supinfo.docx.

## Data Availability

The data supporting the findings of this study are comprehensively detailed within the article itself, rendering additional requests for data unnecessary.
