# Supplementary material for: The Role of Susceptibility in the Association Between Exposures and Occupational Contact Dermatitis: A Scoping Review
Source: Contact Dermatitis. 2025 Sep 12;94(1):1–27. doi: 10.1111/cod.70030 (PMC12695516; doi:10.1111/cod.70030)
Supplement: Supplementary file 1 — Data S1: cod70030‐sup‐0001‐supinfo.docx. [file COD-94-1-s001.docx]

Supplement 1: Search strategies

MEDLINE (Ovid)

Database(s): Ovid MEDLINE(R) and Epub Ahead of Print, In-Process, In-Data-Review & Other Non-Indexed Citations and Daily 1946 to February 19, 2024
Search Strategy:

| # | Searches | Results |
| --- | --- | --- |
| 1 | exp Dermatitis, Contact/ or Hand Dermatoses/ | 42181 |
| 2 | contact dermatitis.ti,ab,kf. | 15947 |
| 3 | (occupational adj2 (dermatitis or eczema)).ti,ab,kf. | 1994 |
| 4 | (allerg* adj2 dermatitis).ti,ab,kf. | 10713 |
| 5 | (irritant* and dermatitis).ti,ab,kf. | 3031 |
| 6 | (eczema and contact).ti,ab,kf. | 2554 |
| 7 | (contact adj12 (sensiti* or hand dermatos* or hand eczema)).ti,ab,kf. | 8660 |
| 8 | 1 or 2 or 3 or 4 or 5 or 6 or 7 | 52564 |
| 9 | exp Occupational Exposure/ or exp Occupations/ or Occupational Health/ or exp Occupational Diseases/ or exp Work/ or exp Workplace/ or exp Occupational Groups/ or exp health personnel/ or exp nurses/ or exp Industry/ | 1309149 |
| 10 | (occupation* or worker* or employee* or industries or industrial sector* or food industry or construction industry or workp* or job or jobs or staff or personnel or hairdresser* or nurs* or cleaner* or painter*).ti,ab,kf. | 1250753 |
| 11 | ((occupation* or work or worker*) adj2 expos*).ti,ab,kf. | 49027 |
| 12 | (irritant* or irritative or phototoxic* or wet work or wet exposur* or detergent* or cutting fluid*).ti,ab,kf. | 68806 |
| 13 | exp Irritants/ or Industrial oils/ae or Trichloroethylene/ae or Solvents/ae or Epoxy Resins/ae or exp Hair Preparations/ae | 19876 |
| 14 | 9 or 10 or 11 or 12 or 13 | 2232697 |
| 15 | exp Disease Susceptibility/ or exp risk/ or causality/ or incidence/ or Prognosis/ or exp epidemiologic factors/ or exp survival analysis/ or exp Life Style/ or (etiology or epidemiology or genetics).fs. | 9840034 |
| 16 | (risk* or predict* or epidemiolog* or etiolog* or genetic* or prognos* or incidence or life style* or lifestyle* or personal factor* or susceptib* or causal* or confound* or mediator* or moderator* or course or survival).ti,ab,kf. | 8865602 |
| 17 | 15 or 16 | 14391123 |
| 18 | 8 and 14 and 17 | 12662 |
| 19 | exp cohort studies/ or exp case-control studies/ or Cross-Sectional Studies/ or (case-control* or control* or cohort* or follow-up or prospectiv* or retrospectiv* or cross sectional*).ti,ab,kf. | 8494158 |
| 20 | 8 and 14 and 17 and 19 | 2630 |
| 21 | (exp Animals/ or exp Animal Experimentation/ or exp models, animal/ or (rat or rats or mice or mouse or murine or murines or rodent or rodents or rabbit or rabbits or cat or cats or dog or dogs or pig or pigs or cow or cows or monkey or monkeys or goat or goats or horse or horses or ape or apes or gorilla or gorillas or sheep or sheeps or ovine or lamb or swine or swines or porcine or pup or pups or canine or beagle).ti,ab,kf.) not Humans/ | 5548809 |
| 22 | 20 not 21 | 2481 |
| 23 | letter/ or comment/ or editorial/ or meta-analysis/ or "systematic review"/ or exp "review"/ or exp Congresses as Topic/ or (letter or comment* or editorial or conference abstract* or conference proceeding* or systematic review or meta-analys* or metaanalys* or review).ti. | 5968701 |
| 24 | 22 not 23 | 2186 |

EMBASE (Ovid)

Database(s): Embase Classic+Embase 1947 to 2024 February 19
Search Strategy:

| # | Searches | Results |
| --- | --- | --- |
| 1 | exp contact dermatitis/ or hand eczema/ or exp occupational eczema/ | 39234 |
| 2 | exp *dermatitis/ and occupational exposure/ | 1465 |
| 3 | contact dermatitis.ti,ab,kf. | 25887 |
| 4 | (occupational adj2 (dermatitis or eczema)).ti,ab,kf. | 2645 |
| 5 | (allerg* adj2 dermatitis).ti,ab,kf. | 17762 |
| 6 | (irritant* and dermatitis).ti,ab,kf. | 5173 |
| 7 | (eczema and contact).ti,ab,kf. | 5188 |
| 8 | (contact adj12 (sensiti* or hand dermatos* or hand eczema)).ti,ab,kf. | 12358 |
| 9 | 1 or 2 or 3 or 4 or 5 or 6 or 7 or 8 | 62793 |
| 10 | occupational exposure/ or exp occupation/ or employee/ or exp work/ or occupational health/ or occupational disease/ or exp occupational allergy/ or exp occupational skin disease/ or exp health care personnel/ or exp nonmedical occupations/ | 3094985 |
| 11 | (occupation* or worker* or employee* or industries or industrial sector* or food industry or construction industry or workp* or job or jobs or staff or personnel or hairdresser* or nurs* or cleaner* or painter*).ti,ab,kf. | 1589215 |
| 12 | ((occupation* or work or worker*) adj2 expos*).ti,ab,kf. | 64708 |
| 13 | (irritant* or irritative or phototoxic* or wet work or wet exposur* or detergent* or cutting fluid*).ti,ab,kf. | 87022 |
| 14 | exp irritant agent/ or exp industrial chemical/ae or trichloroethylene/ae or exp solvent/ae or epoxy resin/ae or hair bleaching agent/ae or hair dye/ae | 23762 |
| 15 | 10 or 11 or 12 or 13 or 14 | 3970134 |
| 16 | genetic susceptibility/ or causality/ or exp risk/ or exp risk assessment/ or prediction/ or exp incidence/ or prognosis/ or survival analysis/ or exp epidemiology/ or exp lifestyle/ or (etiology or epidemiology or genetics).fs. | 10101137 |
| 17 | (risk* or predict* or epidemiolog* or etiolog* or genetic* or prognos* or incidence or life style* or lifestyle* or personal factor* or susceptib* or causal* or confound* or mediator* or moderator* or course or survival).ti,ab,kf. | 12205227 |
| 18 | 16 or 17 | 16414933 |
| 19 | 9 and 15 and 18 | 11983 |
| 20 | cohort analysis/ or exp case control study/ or follow up/ or prospective study/ or retrospective study/ or cross-sectional study/ or major clinical study/ or (case-control* or control* or cohort* or follow-up or prospectiv* or retrospectiv* or cross sectional*).ti,ab,kf. | 13767065 |
| 21 | 9 and 15 and 18 and 20 | 3976 |
| 22 | (exp animal/ or exp animal experiment/ or exp experimental animal/ or exp animal model/ or nonhuman/ or (rat or rats or mice or mouse or murine or murines or rodent or rodents or rabbit or rabbits or cat or cats or dog or dogs or pig or pigs or cow or cows or monkey or monkeys or goat or goats or horse or horses or ape or apes or gorilla or gorillas or sheep or sheeps or ovine or lamb or swine or swines or porcine or pup or pups or canine or beagle).ti,ab,kw.) not human/ | 8527819 |
| 23 | 21 not 22 | 3887 |
| 24 | letter/ or editorial/ or note/ or "systematic review"/ or "review"/ or exp meta analysis/ or (letter or comment* or editorial or conference abstract* or conference proceeding* or systematic review or meta-analys* or metaanalys* or review).ti. | 6707606 |
| 25 | 23 not 24 | 3457 |
| 26 | limit 25 to conference abstract status | 756 |
| 27 | 25 not 26 | 2701 |

CINAHL (EBSCOhost)

506 Results

( (MH "Dermatitis, Contact") OR (MH "Dermatitis, Atopic") ) OR TI ( contact dermatitis OR allergic dermatitis OR occupational dermatitis OR occupational eczema OR hand dermatoses OR irritant dermatitis OR eczema ) OR AB ( contact dermatitis OR allergic dermatitis OR occupational dermatitis OR occupational eczema OR hand dermatoses OR irritant dermatitis OR eczema )

AND

(MH "Occupational Exposure") OR ( (MH "Occupational Health") OR (MH "Occupational Diseases") OR (MH "Work") OR (MH "Work Environment") OR (MH "Health Personnel+") OR (MH "Nurses") OR (MH "Industry") OR (MH "Food Industry+") OR ( TI (occupation* OR worker* OR employee* OR industries OR industrial sector* OR food industry OR construction industry OR workp* OR job OR jobs OR staff OR personnel OR hairdresser* OR nurs* OR cleaner* OR painter* OR irritant* OR irritative OR phototoxic* OR wet work OR wet exposur* OR detergent* OR cutting fluid* OR occupational exposure* OR work exposure* OR workers exposure* OR worker exposure*) OR AB (occupation* OR worker* OR employee* OR industries OR industrial sector* OR food industry OR construction industry OR workp* OR job OR jobs OR staff OR personnel OR hairdresser* OR nurs* OR cleaner* OR painter* OR irritant* OR irritative OR phototoxic* OR wet work OR wet exposur* OR detergent* OR cutting fluid* OR occupational exposure* OR work exposure* OR workers exposure* OR worker exposure*) )

AND

(MH "Disease Susceptibility+") OR (MH "Risk Factors") OR (MH "Risk Assessment") OR (MH "Prognosis") OR (MH "Predictive Research") OR (MH "Incidence") OR (MH "Epidemiology") OR (MH "Life Style+") OR (MH "Survival Analysis") OR ( (MH "Causality") OR (MH "Confounding") ) OR ( TI (risk* OR predict* OR epidemiolog* OR etiolog* OR genetic* OR prognos* OR incidence OR life style* OR lifestyle* OR personal factor* OR susceptib* OR causal* OR confound* OR mediator* OR moderator* OR course OR survival) OR AB (risk* OR predict* OR epidemiolog* OR etiolog* OR genetic* OR prognos* OR incidence OR life style* OR lifestyle* OR personal factor* OR susceptib* OR causal* OR confound* OR mediator* OR moderator* OR course OR survival))

Supplement 2: Selection criteria for title and abstract screening and full-text screening

| **Inclusion criteria** | **Exclusion criteria** |
| --- | --- |
| **For the population:**  We will include studies with explicit identification of workers or occupations (e.g., healthcare workers, hairdressers, construction workers, and painters), apprentices, industries (e.g., car or food) or the general population exposed to workplace irritants/allergens that are known to be associated with the outcome (see Appendix B in the published protocol for examples of allergens/irritants^19^).  If the study population consists of both the working population and the non-working population, the analyses should be stratified for the working population (so that we can use data of workers only in our synthesis). | Studies on:   - The general population without a clear occupational exposure (i.e., irritants/allergens at home or in non-work environments); - Non-employed individuals (i.e., children, elderly); - Workers lacking exposure to irritants/allergens that are known to be associated with the outcome (see Appendix B in the published protocol for examples of allergens/irritants^19^). |
| **For the outcome:**  Studies on the occurrences of (contact) dermatitis (ICD and/or ACD)  Eligible studies include those with clinical assessments or those with less diagnostic assessment (e.g., self-reports). | Studies with the following outcomes:   - Atopic dermatitis (AD) - Contact sensitization/allergy   (n.b. frequently identified through **patch tests** conducted on either **healthy** **individuals** or patients)   - Other types of skin diseases ( i.e., contact urticaria (CU), protein contact dermatitis (PCD), photoallergic contact dermatitis (PACD), photo irritant contact dermatitis (PICD)) - Types of dermatitis not related to work (i.e., incontinence dermatitis) - Dermatosis (without a specific mentioning of CD) |
| **For the individual susceptibility factor:**  TIAB screening  No requirements need to be met, even if there are **no indications** of individual susceptibility factors, studies would still be included for full-text screening.  Full-text  Quantified effect estimates (e.g., PR, OR,RR, HR) of one or more personal (e.g. sex, age, genetic factors, and allergic comorbidities) and lifestyle factors (e.g. smoking and alcohol consumption) that can mediate, moderate, and/or confound the effect of exposure to irritants and/or allergens and CD.  We include articles that present with unadjusted and adjusted estimates (i.e., through mediation, moderation, or confounding, see Figure 1 in the published protocol^19^) for each individual susceptibility factor on the association between the irritant or allergen exposure and CD. | TIAB screening  Non applicable  Full-text  Studies that do not include individual susceptibility factors that can influence the association between the irritant or allergen exposure and diagnosed CD within the working population.  Studies reporting that the analyses for an exposure and CD have been adjusted for one or more individual susceptibility factor **without explicitly** presenting the estimates of these factors with adjusted and unadjusted estimates (i.e., through mediation, moderation, or confounding, see Figure 1 in the published protocol^19^) for each individual susceptibility factors. |
| **Study designs**:  TIAB-screening  Cohort, retrospective and prospective studies, case-control, case-referent and cross-sectional studies.  Full-text screening  In addition to the above mentioned criteria for TIAB-screening, cross-sectional studies will **only** be included when associated with the following outcomes:   - ACD - CD induced by contact allergens   (see Appendix B in the published protocol for examples of allergens/irritants^19^)   - Allergic eczema - Confirmation of ACD via patch testing | TIAB-screening  Reviews (e.g., literature, systematic and scoping), letters to the editor, conference abstracts, experimental studies (i.e., quasi-experimental studies and RCT), case reports (and case series), qualitative studies, and animal studies.  Full-text screening  In addition to the above mentioned criteria for TIAB-screening, we will exclude cross-sectional studies associated with either ICD or CD/eczema induced by irritants (see Appendix B in the published protocol for examples of allergens/irritants^19^) |
| Publication in a peer-reviewed journal, available in either English or Dutch. | Publications in non-peer-reviewed journals or in languages other than English or Dutch. |

**Abbreviations**: CD= contact dermatitis, ICD= irritant contact dermatitis, ACD= allergic contact dermatitis, TIAB = title and abstract screening, PR= prevalence ratio, OR= odds ratio, RR= relative risk, HR= hazard ratio.
